# A Fixed-Dose Combination of Ofloxacin-Ornidazole Induced Fixed Drug Eruption: A Case Report

**DOI:** 10.7759/cureus.35630

**Published:** 2023-03-01

**Authors:** Manuj K Sarkar, Subhra Dey

**Affiliations:** 1 Internal Medicine, All India Institute of Medical Sciences, Deoghar, Deoghar, IND; 2 Dentistry, All India Institute of Medical Sciences, Deoghar, Deoghar, IND

**Keywords:** hyperpigmented drug reaction, hyperpigmented linear rash, ofloxacin-ornidazole, cutaneous drug eruption, fixed dose combination, fixed drug eruption

## Abstract

Cutaneous drug eruptions are commonly occurring adverse drug reactions. Food and Drug Association does not recommend a fixed dose combination of ofloxacin-ornidazole; still, it is commonly practised in developing countries. Many patients take this combination of drugs for episodes of gastro-enteritis, often as self-medication. We are reporting a 25 years old male patient presenting with repeated episodes of adverse drug reaction to a fixed dose combination of ofloxacin-ornidazole.

## Introduction

Cutaneous adverse drug reactions (ADR) are distinct, sharply defined lesions, oval to rounded, can be single or multiple, usually seen in the extremities, genitals, or perianal area, but any part of the body can be affected [1-3]. We observe skin lesions typically 1-10 hours after exposure; redness and swelling appear first and can be associated with blisters, which can persist for a few weeks. Later healing occurs with residual hyperpigmentation [2,3]. Cutaneous drug eruptions are the most common type of drug reaction. Usually, they are self-limiting. They are called fixed drug eruptions (FDE). FDE occurs in 8% of the global population and 2-3% of all hospital admissions [4]. Common drugs causing FDE are antibiotics, anticonvulsants, antivirals, and non-steroidal anti-inflammatory drugs (NSAIDs). We can see FDE in all age groups, the most common age group being 20-40 years [1]. FDE lesions first appear when a susceptible person is sensitized to the particular drug. This sensitization occurs slowly who take the drug intermittently. Intra-epidermal CD8+ T cells with an effector memory phenotype are found in abundance in FDE lesions. These T cells express TCR, CD3, CD8, CD45RA, and CD11, which resemble memory T cells. Once activated, intradermal CD8+ T cells show cytolytic activity by the CD3-TCR complex, thus killing basal keratinocytes and releasing many cytokines. Memory T cells are capable of re-start the previous reaction and thus delayed type IV hypersensitivity reaction is maintained at the site of the previous lesion [5]. Repeated exposure to the offending agent causes an increase in hyperpigmentation of the skin by deposition of melanin, and sometimes different drugs of the same group cross-react with memory cells of the previously sensitized basal cells of the skin and produce similar lesions. Drug act as a hapten and bind to the skin’s basal keratinocytes and start hypersensitivity reactions [2].

Ornidazole is a drug of nitro-imidazole group antibiotic, having excellent activity against anaerobic organisms and protozoa. Ofloxacin belongs to second-generation fluoroquinolone and has activity against bacteria. A fixed-dose combination (FDC) of ofloxacin and ornidazole is inappropriate, it increases the chances of interaction and side effects of drugs, and standard books and guidelines do not recommend it. A case of pancreatitis is also being reported with this combination, but injudicious use of such FDC of drugs is widely available in India and commonly prescribed by physicians and also it is taken as self-medication [6-8].

We are reporting a typical case of Fixed Drug Eruption induced by the irrational use of FDC of ofloxacin and ornidazole as self-medication.

## Case presentation

**Figure 1:**
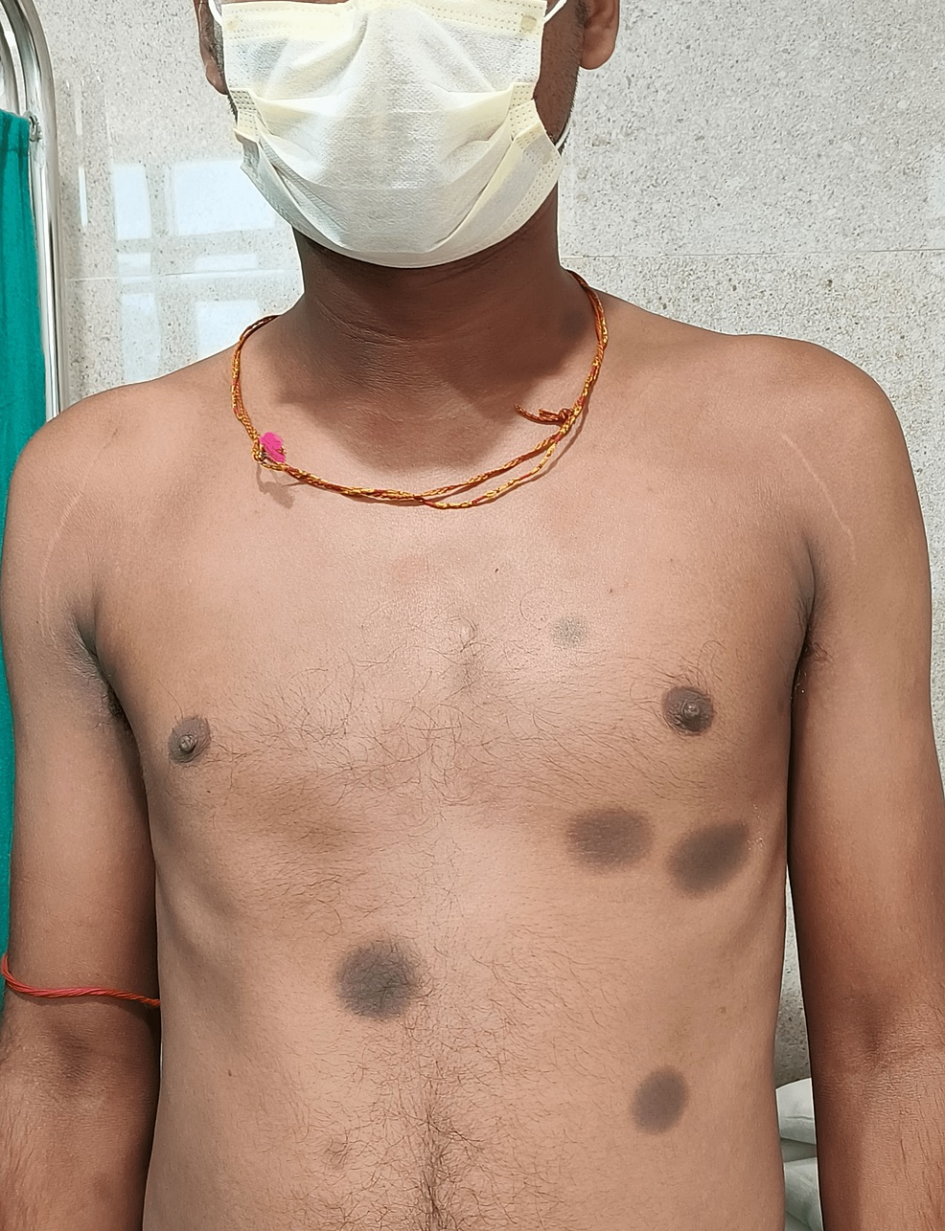
Multiple hyperpigmented, sharply defined lesions in the anterior aspect of chest and abdomen

**Figure 2:**
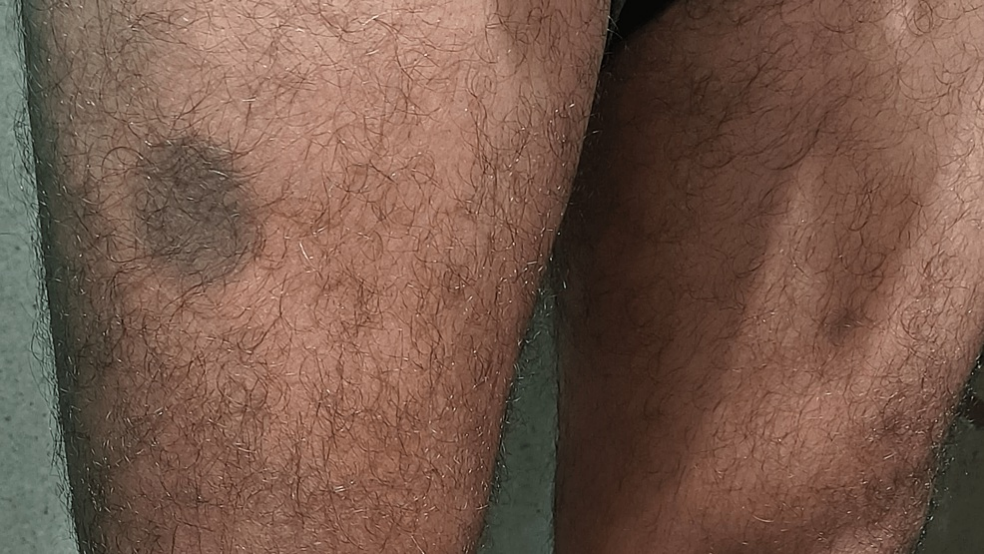
Hyperpigmented lesion in anterior aspect of right thigh

A 25-year-old male patient presented with a history of repeated episodes of skin eruptions following self-medication of a fixed-dose combination of tablets ofloxacin-200 mg and ornidazole-500 mg. He noticed the first episode two years back when he took the offending drug for an episode of acute gastroenteritis (AGE). During the first episode, the lesions were erythematous, violaceous, circular to oval, flat-topped, and rounded maculopapular, involving the anterior aspect of the chest, upper abdomen, and anterior part of the thigh. He consulted a local practitioner, who prescribed oral anti-histaminic and topical corticosteroid ointment. Initially, the patient was not aware that the lesions were caused by the drug he was taking. Later he noticed similar lesions used to appear in the same part of the body, which persist for a few days to weeks and subsides with residual hyperpigmentation of the affected part, which was clearly demarcated from the surrounding skin (Figures 1,2). He also gave a history of a few newer lesions besides old hyperpigmented lesions.

**Figure 3:**
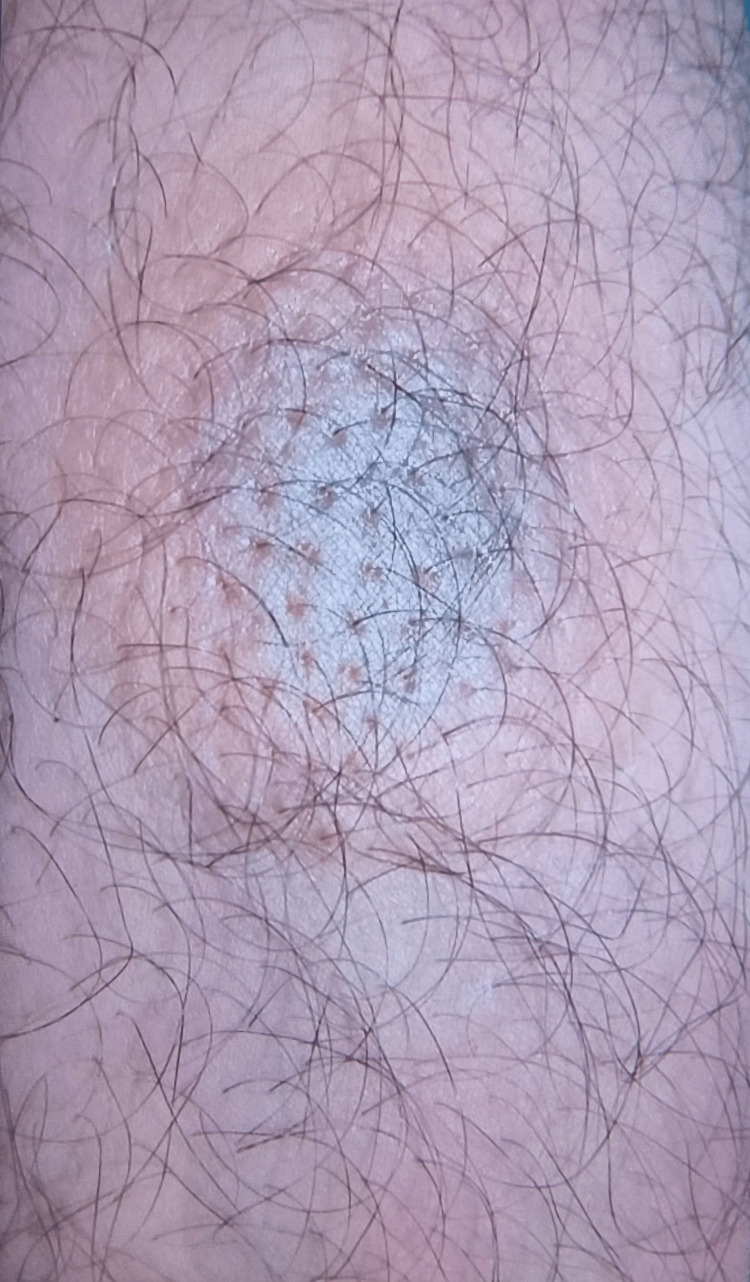
New lesion on antero-lateral aspect of left thigh with central hyperpigmentation and peripheral skin edema

The latest episode developed one week prior to presentation to out patient department (OPD) of All India Institute of Medical Sciences, Deoghar, when he himself took FDC of two tablets of ofloxacin 200 mg and ornidazole 500 mg for one day for an episode of AGE. In fact, he visited our hospital to consult for alternative medicine for treatment of AGE. He noticed in the evening of the same day after consumption of the drug that a new lesion appeared on the antero-lateral part of left thigh; it showed a central violaceous, hyperpigmented area with surrounding redness and swollen skin of 1.5x1.5 cm^2^ size (Figure 3). He also noticed that the previous lesions became more pigmented compared with the previous residual lesions, along with itching at the lesions.

There was no history of any skin disease, hypertension, diabetes mellitus, no history of autoimmune disease, connective tissue disease, no history of allergy to food or any other drugs. Though he was aware of the agent causing his condition, he still preferred to consume the offending drug. His vital examinations were within normal range. He did not have any other abnormality. He was reassured and advised not to use a similar combination of drugs in the future. We also explained the dangerous situations which may arise from self-medications. We advised him to follow instructions from a registered medical practitioner.

## Discussion

Self-medication is a very common practice in developing countries like India. Many people used to take a fixed-dose combination of ofloxacin-ornidazole for acute gastroenteritis. Self-medication is defined as “the taking of drugs, herbs or home remedies on one’s own initiative, or on the advice of another person, without consulting a doctor” [9].

Our patient also took the fixed-dose combination of ofloxacin-ornidazole as self-medication. Initially, he was not aware of the consequences, but gradually with repeated episodes, he became aware and when he visited our OPD, he himself gave a history of drug eruption. His hyperpigmented lesions were because of the offending drug. Repeated exposure to the same drug/drugs or pharmacologically similar drugs can cause cross-reactions and cause similar lesions [10].

We can see several forms of FDE in a single patient. It can be pigmented, non-pigmented, or bullous FDE [11]. Our patient presented this time with swollen lesions, which later turned pigmented, and his lesions showed a typical evolution of FDE with repeated exposure to the same offending drug. Though the exact mechanism for this kind of reaction is not known. The drugs start an inflammatory process by binding with basal keratinocytes, which causes the release of inflammatory granules, interferon, and lymphokines. Repeated exposure causes stimulation of mast cells locally and starts an inflammatory reaction each time we expose the person to the offending drugs [2,5-8,11,12].

Though the FDC of ofloxacin and ornidazole is inappropriate, it is commonly available without prescription and used in developing countries [3,6-8]. Chakrabarti A. studied the proportion of physicians using the FDC of antiprotozoal and antibacterial agents for diarrhoeal disease from India. He found that out of 2163 physicians’ prescriptions, 59% were prescribing FDC of the fixed-dose combinations of ofloxacin and ornidazole [13].

## Conclusions

FDE caused by FDC of antiprotozoal and antibacterial medication like ofloxacin-ornidazole and other related compounds are very common in developing countries, though it is not reported. As this combination does not provide any added benefit and rather increases the adverse effects, including FDE, combined preparation can be avoided by prescribing individual drugs separately when they are required. Thus, the incidence of fixed drug eruptions can be reduced. Policymakers and administrators must take measures to reduce the availability of such drugs. Patients and physicians need to be educated regarding the pros and cons of such FDC of drugs, which are available commercially, and judicious use of these drugs only when they are extremely required.
